# Associations of systemic inflammatory indices and metabolically healthy vs. unhealthy obesity in children

**DOI:** 10.1186/s13052-026-02262-1

**Published:** 2026-04-15

**Authors:** Meliha Esra Bіlіcі

**Affiliations:** Department of Pediatric Endocrinology, Zonguldak Bulent Ecevit University Faculty of Medicine, İbni Sina Campus, Zonguldak, 67600 Turkey

**Keywords:** Childhood obesity, Metabolic healthy obesity (MHO), Metabolic unhealthy obesity (MUO), Systemic immune-inflammation index (SII), Systemic immune-inflammation response index (SIRI)

## Abstract

**Objective:**

Childhood obesity, which continues to rise globally, is associated with inflammation-driven metabolic disturbances and increased cardiometabolic risk. Individual variability in metabolic health—independent of obesity severity—has led to the concept of metabolically healthy obesity (MHO). This study aimed to evaluate the relationship between metabolic health status and systemic inflammatory indices in childhood obesity, including analyses adjusted for sex and pubertal stage to account for physiologic developmental changes.

**Methods:**

This retrospective study included children with BMI SDS ≥ 2, categorised as Metabolically Unhealthy Obese (MUO) if they had ≥ 1 of the following: fasting glucose ≥ 100 mg/dL, HOMA-IR ≥ 2.5 (prepubertal) or ≥ 4 (pubertal), triglycerides ≥ 150 mg/dL, HDL ≤ 40 mg/dL, or systolic/diastolic blood pressure ≥ 95th percentile. Children with none of these findings were classified as MHO. Inflammatory indices—neutrophil-lymphocyte ratio (NLR), platelet-lymphocyte ratio (PLR), systemic inflammation index (SII), and systemic immune-response index (SIRI)—were analysed using ROC curves and multivariable regression. Additional analyses adjusted for age, sex, pubertal stage, and BMI SDS were performed to account for developmental influences on inflammatory markers.

**Results:**

A total of 388 obese children (58.1% female, mean age 11.5 ± 2.9 years) were included; 35.4% were morbidly obese, and 45.6% were classified as MUO. MUO children had significantly higher white blood cell, neutrophil, and monocyte counts, as well as higher fasting glucose, HOMA-IR, triglycerides, and total cholesterol, with lower HDL levels. Dyslipidemia was the most common metabolic abnormality. SII demonstrated strong discriminatory ability for metabolic health (AUC = 0.854; 82% sensitivity; 72% specificity). SII was positively associated with HOMA-IR, TG/HDL-C, and non-HDL cholesterol (*p* = 0.006; *p* = 0.004; *p* = 0.01, respectively). After adjustment for age, sex, pubertal stage, and BMI SDS, the associations of SII and SIRI with metabolic health status were attenuated but remained directionally consistent, indicating that developmental factors partially influence systemic inflammatory responses.

**Conclusion:**

The high prevalence of MUO among obese children highlights their substantial cardiometabolic risk burden. SII and SIRI appear to be promising, readily accessible biomarkers for identifying metabolic deterioration, showing meaningful correlations with key cardiometabolic risk indicators. These indices may support clinicians in early detection and monitoring of metabolic dysfunction in pediatric obesity, even when accounting for developmental stage.

## Introduction

Childhood obesity has emerged as a major global health concern due to its rapidly increasing prevalence and well-documented long-term metabolic and cardiovascular consequences. Beyond excessive adiposity, the heterogeneity of metabolic responses among obese individuals has heightened interest in the concept of metabolic health. Obese children are at increased risk for dyslipidemia, insulin resistance, hypertension, and early cardiovascular remodeling, and current guidelines recommend that each component of metabolic syndrome (MetS) be managed as an independent cardiometabolic risk factor [1–3].

Although more than 40 definitions of MetS have been proposed—many of them extrapolated from adult criteria—the observation that a subset of obese individuals display a markedly attenuated cardiometabolic risk led to the recognition of the “metabolically healthy obesity” (MHO) phenotype. MHO refers to obesity accompanied by preserved metabolic parameters, whereas metabolically unhealthy obesity (MUO) describes obesity with abnormalities such as insulin resistance, hyperglycemia, dyslipidemia, and hypertension [4, 5]. Emerging evidence suggests that MHO may be associated with lower visceral and hepatic fat accumulation, greater subcutaneous adipose tissue expandability, preserved β-cell function, and enhanced insulin sensitivity [5–8]. However, the mechanistic distinctions between these phenotypes remain incompletely understood, and chronic low-grade inflammation has been proposed as a key biological determinant.

Metabolic inflammation is a chronic, low-grade inflammatory process triggered by excessive nutrient accumulation within adipocytes. In obesity, adipocyte hypertrophy and energy surplus induce homeostatic stress and activate chemokine-mediated adaptive inflammatory pathways. Once the adipose tissue expandability threshold is exceeded, this response transitions into a persistent inflammatory cycle. Circulating pro-inflammatory mediators—such as TNF-α, IL-6, and acute-phase reactants—increase in proportion to adipose tissue dysfunction, positioning adipose tissue as the central regulator of metabolic inflammation [3, 8–12].

The role of inflammation in pediatric obesity was first highlighted by Cook et al., who reported elevated C-reactive protein levels in obese youth [13]. Subsequent studies consistently demonstrated increases in pro-inflammatory cytokines such as TNF-α and IL-6, accompanied by reduced adiponectin levels, all contributing to adverse metabolic outcomes [4, 13–16]. Low-grade systemic inflammation originating from adipose tissue has also been associated with early cardiovascular alterations, including left ventricular hypertrophy and increased carotid intima–media thickness in children [17–19]. Puberty, moreover, represents a physiologic period of immunoendocrine activation characterized by transient elevations in inflammatory markers, complicating the interpretation of metabolic inflammation in adolescents.

Recently, hematological indices derived from complete blood count—such as the neutrophil-to-lymphocyte ratio (NLR), platelet-to-lymphocyte ratio (PLR), systemic immune-inflammation index (SII), and systemic inflammation response index (SIRI)—have gained attention as accessible and cost-effective biomarkers reflecting systemic inflammation and endothelial dysfunction. However, evidence regarding their utility in distinguishing metabolic phenotypes in pediatric obesity remains limited [9–11, 20–22]. Most available studies focus on adult cohorts, and research assessing these inflammatory hematologic markers in differentiating MHO and MUO among children and adolescents is extremely scarce [10, 12]. The lack of data on their discriminative value—particularly during puberty, when physiological inflammation is naturally amplified—represents a significant gap in the current literature.

The primary aim of this study is to evaluate the utility of hematological inflammatory indices in distinguishing metabolically healthy from metabolically unhealthy obesity in children. A secondary aim is to examine the associations between these indices and key cardiometabolic risk markers, taking into account the physiological inflammatory changes accompanying pubertal development.

## Methods

The study retrospectively reviewed the medical records of children and adolescents aged 0–18 years who presented to the Pediatric Endocrinology Clinic of Zonguldak Bülent Ecevit University between April 2021 and December 2024, in accordance with the principles of the Declaration of Helsinki. Ethical approval was obtained from the Zonguldak Bülent Ecevit University Clinical Research Ethics Committee (approval date: April 30, 2025; approval number: 2025/09). Owing to the study’s retrospective design, the ethics committee waived the requirement for informed consent.

Children with a BMI-SDS ≥ 2 were included in the study, and those with a BMI-SDS ≥ 2 were classified as obese, whereas children with a BMI-SDS ≥ 3 were defined as morbidly obese. Children with syndromic or hypothalamic obesity, those receiving any pharmacological treatment, or those with accompanying autoimmune diseases, active infections, or other chronic conditions were excluded.

Anthropometric measurements, triglyceride, high-density lipoprotein cholesterol [HDL], low‐density lipoprotein cholesterol [LDL], total cholesterol, fasting blood sugar, fasting insulin level, alanine transaminase [ALT], aspartate transaminase [AST], thyroid-stimulating hormone [TSH], free T4 level [FT4] and complete blood count parameters of the patients taken at the time of admission were recorded. TSH and FT4 levels were recorded to exclude possible thyroid dysfunction, as changes in thyroid status may affect metabolic health-related parameters and act as potential confounders in the assessment of metabolic and inflammatory indices. Biochemical parameters were measured by the spectrophotometric method with a chemical autoanalyser, while hormone levels [TSH, FT4] were measured by the electrochemiluminescence method [Cobas 6000 (Roche Diagnostics, Mannheim, Germany)]. The Homeostatic Model Assessment for Insulin Resistance (HOMA-IR) was calculated using the formula: fasting glucose (mg/dL) × fasting insulin (µIU/mL) / 405 [20, 21].

Pubertal staging was assessed according to Tanner criteria. Girls were classified based on breast development, while in boys, pubertal status was determined using testicular volume measured with a Prader orchidometer. Boys with a testicular volume of < 4 mL were considered prepubertal (Tanner stage 1), those with volumes between 4 and 10 mL, 10–15 mL, and 15–20 mL were classified as Tanner stages 2, 3, and 4, respectively, and volumes between 20 and 25 mL were accepted as postpubertal (stage 5). For analytical purposes, participants of both sexes were categorized into three groups: prepubertal (stage 1), pubertal (stages 2–4), and postpubertal (stage 5). Pubertal stage and sex were included as covariates in the statistical analyses to control for their potential confounding effects on inflammatory indices.

The cases were categorised into two groups according to metabolic health parameters. The Metabolically Unhealthy Obese cohort comprised individuals exhibiting one or more metabolic risk factors, including fasting glucose levels ≥ 100 mg/dL, HOMA-IR values ≥ 2.5 [prepubertal] or ≥ 4 [pubertal], triglycerides ≥ 150 mg/dL, HDL levels ≤ 40 mg/dL, and systolic and diastolic blood pressure at or above the 95th percentile. The MHO group comprises individuals with normal metabolic health criteria [22].

Complete blood count parameters were studied for both groups with the Sysmex XN-550 automated system [Sysmex Corporation, Kobe, Japan]. The neutrophil/lymphocyte ratio [NLR], platelet/lymphocyte ratio [PLR], systemic immune-inflammation index [SII] (platelet × neutrophil/lymphocyte), and systemic immune response index [SIRI] (monocyte × platelet/lymphocyte) were calculated from these parameters to assess the inflammatory status. The reliability of the inflammatory parameters as indicators of metabolic health was evaluated using the Receiver Operating Characteristic [ROC] analysis to determine the threshold values. Additionally, the associations between inflammatory indices and cardiometabolic risk variables, including HOMA-IR, triglyceride/HDL ratio, and non-HDL cholesterol, were assessed by multivariable regression analysis.

## Statistical analysis

Statistical analyses were performed using SPSS version 19.0 (IBM Corp., Armonk, NY, USA). The Shapiro–Wilk test was used to evaluate the normality of continuous variables. Parametric data were analysed using the independent samples t-test, while non-parametric data were compared using the Mann–Whitney U test. Categorical variables were analysed with the chi-square or Fisher’s exact test where appropriate. Continuous variables were presented as mean ± standard deviation or median [minimum–maximum], and categorical variables as percentages.

To account for the physiological effects of puberty on inflammatory markers, pubertal stage was included as a covariate in multivariable analyses. Group comparisons of inflammatory indices (SII, SIRI, NLR, PLR) between MHO and MUO were performed using General Linear Models (GLM) adjusted for age, sex, BMI SDS, and pubertal stage, yielding puberty-adjusted mean values.

ROC analyses were conducted to assess the diagnostic performance of systemic inflammatory indices in distinguishing MHO from MUO. The area under the curve (AUC), standard error (SE), and 95% confidence intervals (95% CI) were calculated. Optimal cut-off values were determined using the Youden index.

To examine the associations between inflammatory indices and cardiometabolic risk parameters (HOMA-IR, triglyceride/HDL ratio, and non-HDL cholesterol), multivariable linear regression analyses were performed. Regression coefficients (β), R² values, 95% CI, and p-values were reported.

Additionally, to explore potential differences by sex and pubertal stage, an age-adjusted two-way ANOVA was conducted with metabolic phenotype (MHO vs. MUO) and sex/pubertal status as fixed factors. Interaction terms were examined, and post-hoc pairwise comparisons were applied when appropriate. A p-value < 0.05 was considered statistically significant. Statistically significant values in tables were highlighted in bold and italics.

## Results

A total of 388 children with obesity were included in the analysis. The mean age of the cohort was 11.5 ± 2.9 years, with no significant age difference between the MHO and MUO groups (11.2 ± 3.4 vs. 11.7 ± 3.05 years, *p* = 0.980). Overall, 35.4% (*n* = 137) of the participants were classified as morbidly obese. Morbid obesity was significantly more prevalent in the MUO group compared with the MHO group (47.5% vs. 34.0%, *p* = 0.004). Although girls constituted a greater proportion of the MHO group (63.7% vs. 51.1%), the difference did not reach statistical significance (*p* = 0.062).

Anthropometric assessments showed that height-SDS was slightly but significantly lower in MUO children compared with MHO children (*p* = 0.043). In contrast, BMI-SDS values were markedly higher in the MUO group (2.86 ± 1.66) than in the MHO group (2.42 ± 0.7), demonstrating a significant association between higher BMI severity and metabolic unhealthiness (*p* < 0.001). Waist circumference SDS did not differ significantly between groups (*p* = 0.189). Regarding central adiposity, waist circumference did not differ significantly between the groups (94.1 ± 15.4 vs. 93.2 ± 12.2 cm, *p* = 0.805). Similarly, the waist-to-height ratio showed no significant difference (0.48 ± 0.26 vs. 0.45 ± 0.29, *p* = 0.612).

Regarding hematological parameters, MUO children demonstrated significantly higher white blood cell and neutrophil counts compared with the MHO group (*p* < 0.001 and *p* = 0.043, respectively). Monocyte levels were also higher in MUO children (*p* = 0.014). In contrast, lymphocyte and platelet counts did not differ significantly between the groups.

Biochemical assessments revealed notable metabolic differences. MUO children had significantly higher fasting blood glucose levels than MHO children (91.7 ± 11.7 vs. 85.3 ± 25.1 mg/dL, *p* = 0.015). HOMA-IR values were more than double in the MUO group (5.6 ± 3.7 vs. 2.5 ± 1.49, *p* = 0.001), indicating markedly increased insulin resistance. Triglyceride levels were substantially elevated in MUO children (147.9 ± 59.1 vs. 92.7 ± 29.7 mg/dL, *p* < 0.0001), and total cholesterol levels were also significantly higher (156.6 ± 37.5 vs. 145.9 ± 34.7 mg/dL, *p* = 0.016).

Conversely, HDL cholesterol was significantly lower among MUO children (41.4 ± 9.6 vs. 50.0 ± 9.0 mg/dL, *p* < 0.0001). The TG/HDL ratio—an important atherogenic indicator—was nearly twice as high in MUO children (3.6 ± 1.7 vs. 1.8 ± 0.78, *p* < 0.0001). Non-HDL cholesterol levels were also markedly elevated in the MUO group (132 ± 41 vs. 106 ± 24 mg/dL, *p* < 0.001). LDL cholesterol showed a numeric increase in MUO but did not reach statistical significance (*p* = 0.075). There were no significant differences between groups in TSH levels (*p* = 0.284), AST (*p* = 0.064), or ALT levels (*p* = 0.057) (Table 1).

**Table 1:** Comparison of anthropometric, biochemical, and hematological parameters according to metabolic health status

	Total (*n* = 388)	Metabolically Healthy Obese ( *n* = 212)	Metabolically Unhealthy Obese ( *n* = 176) (%45.6)	*p*-value
**Anthropometric Parameters**				
Age (years)	11,5 ± 2,9	11,2 ± 3,4	11,7 ± 3,05	0,980
Morbidly Obese %,(n)	35,4 (137)	34 (72)	47,5 (83)	***0***,***004***
Female Gender %, (n)	58,1 (225)	63,7 (135)	51,1 (90)	0,062
VA SDS	2,8 ± 1,07	2,7 ± 1,06	2,9 ± 1,07	0,189
Height SDS	1,3 ± 1,2	1,6 ± 0,9	1,2 ± 1,1	***0***,***043***
BMI SDS	2,6 ± 0,69	2,42 ± 0,7	2,86 ± 1,66	***< 0***,***001***
WC	93,1 ± 12,9	93,2 ± 12,2	94,1 ± 15,4	*0*,*805*
WC/Height	0,46 ± 0,28	0,45 ± 0,29	0,48 ± 0,26	*0*,*612*
**Hematological Parameters**				
White Blood Cells (×10³/mm³)	4,4 ± 7,4	3,9 ± 6,6	5,32 ± 6,5	< 0,001
Neutrophils (×10³/mm³)	2,1 ± 2,5	1,95 ± 2,48	2,43 ± 2,63	***0***,***043***
Lymphocytes (×10³/mm³)	1,3 ± 1,4	1,17 ± 1,4	1,47 ± 1,47	*0*,*660*
Platelets (×10³/mm³)	159,5 ± 171,6	156 ± 170	177 ± 176,5	*0*,*420*
Monocytes (×10³/mm³)	0,9 ± 0,5	0,25 ± 0,2	0,8 ± 0,6	***0***,***014***
**Biochemical Parameters**				
Fasting Blood Glucose (mg/dL)	85,9 ± 24,4	85,3 ± 25,1	91,7 ± 11,7	***0***,***015***
Insulin (uIU/mL)	21,4 **±** 1,8	22,6 ± 2,1	23,1 ± 2,2	*0*,*412*
HOMA-IR	3,7 ± 3,4	2,5 ± 1,49	5,6 ± 3,7	***0***,***001***
Triglycerides (mg/dL)	113 ± 59	92,7 ± 29,7	147,9 ± 59,1	***< 0***,***0001***
Total Cholesterol (mg/dL)	143,5 ± 48,9	145,9 ± 34,7	156,6 ± 37,5	***0***,***016***
HDL (mg/dL)	43,6 ± 14,3	50,0 ± 9,0	41,4 ± 9,6	***< 0***,***0001***
LDL (mg/dL)	80,4 ± 32,1	82,1 ± 26	88 ± 26,5	*0*,*075*
TG/HDL	2,4 ± 1,7	1,8 ± 0,78	3,6 ± 1,7	***< 0***,***0001***
Non-HDL (mg/dL)	118 ± 16	106 ± 24	132 ± 41	*< 0*,*001*
TSH (mIU/mL)	3,1 ± 1,8	3,0 ± 1,86	3,2 ± 1,76	*0*,*284*
AST (U/L)	23,8 ± 19,6	24,5 ± 17	27 ± 22,5	*0*,*064*
ALT (U/L)	22,5 ± 10,4	21,5 ± 10	23,9 ± 11,2	*0*,*057*

Independent sample *t*-test and Mann-Whitney U Test. Data are expressed as mean±standard deviation, median [minimum, maximum], and percentage [%]. Statistically significant differences are indicated in bold and italicized with a p-value < 0.05. *Abbreviations*: BMI, body mass index; SDS, standard deviation score; WC: Waist circumference; HDL-C, high-density lipoprotein cholesterol; LDL-C, low-density lipoprotein cholesterol; HOMA-IR, homeostasis model assessment of ınsulin resistance; TG: HDL-C, triglyceride-to-high-density lipoprotein cholesterol; non-HDL-C, non-high-density lipoprotein cholesterol. Statistically significant differences between the MHO and MUO groups, with a probability value of *p* < 0.05, are shown in bold

**Fig. 1:**
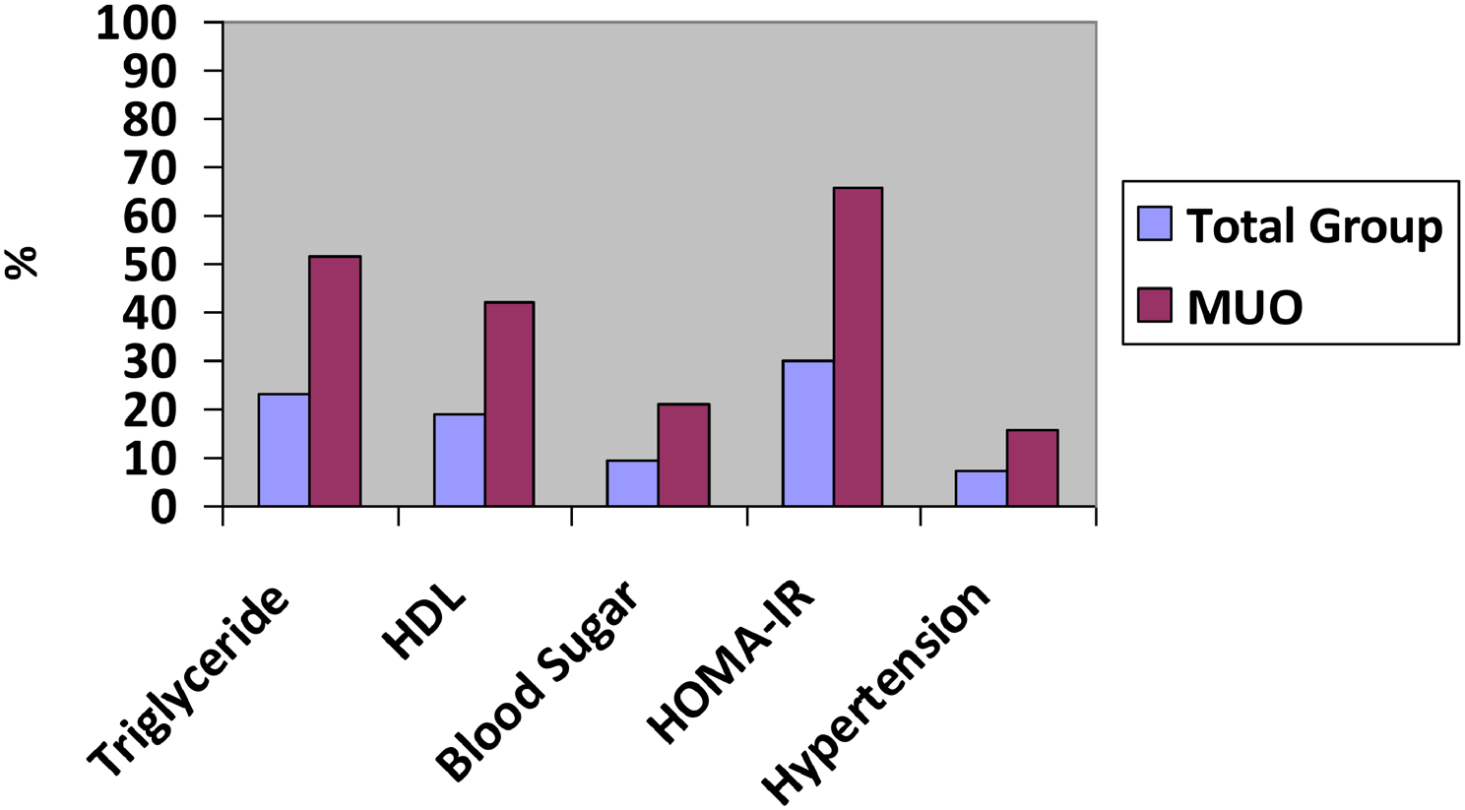
Prevalence of each metabolic health component in the metabolically unhealthy group and the entire study group

Dyslipidemia was found to be the most common cause of metabolic unhealthiness. No differences were observed in metabolic tests among the groups categorised as metabolically unhealthy due to disturbances of glucose metabolism, lipid metabolism or hypertension. In the MUO group, the most common metabolic health deterioration was elevated HOMA-IR [65.9%], followed by elevated triglyceride [51.7%], low HDL [42%], and elevated blood sugar [21%]. The least common component was hypertension, which was detected in 15.9% of children (Fig. 1).

Analysis of hematological inflammatory indices showed that NLR and PLR values did not differ significantly between the groups (*p* = 0.312 and *p* = 0.198, respectively). In contrast, both SII and SIRI demonstrated significant elevations in MUO children. The mean SII value was markedly higher in the MUO group compared with the MHO group (198.1 ± 90.3 vs. 113.5 ± 50.2, *p* = 0.031). Similarly, SIRI values were substantially elevated among MUO participants (1.61 ± 3.4 vs. 0.49 ± 0.7, *p* = 0.024). These findings suggest that SII and SIRI, but not NLR or PLR, may have discriminatory value in identifying metabolically unhealthy obesity (Table 2).

**Table 2:** Hematological inflammatory parameters based on metabolic health status

	Metabolically Healthy Obese (*n* = 212)	Metabolically Unhealthy Obese (*n* = 176)	*p*-value
NLR	0,83 ± 0,92	1,09 ± 1,1	0.312
PLR	60,5 ± 20,2	73,1 ± 46,2	0.198
SII	113,5 ± 50,2	198,1 ± 90,3	***0.031***
SIRI	0,49 ± 0,7	1,61 ± 3,4	***0.024***

Independent sample *t*-test. Data are expressed as mean±standard deviation. Statistically significant differences are indicated in bold and italicized with a p-value < 0.05. *Abbreviations*: SII, systemic immune-inflammation index; NLR, neutrophil-to-lymphocyte ratio; PLR, platelet-to-lymphocyte ratio; SIRI, systemic immune-inflammation response index. Statistically significant differences between MHO group and MUO group, with a probability value of *p* < 0.05, are represented in bold

ROC analysis demonstrated that SII had the strongest discriminatory performance for identifying metabolically unhealthy obesity. SII yielded an AUC of 0.854 with an optimal cut-off value of 146.4, providing 82% sensitivity and 72% specificity (95% CI: 0.770–0.962). In comparison, the SIRI index showed a more modest discriminative ability, with an AUC of 0.675 and a cut-off value of 1.25, corresponding to 72% sensitivity and 64% specificity (95% CI: 0.727–0.893). These results indicate that SII outperforms SIRI as a predictor of metabolic unhealthiness in obese children (Table 3).

**Table 3:** ROC curve analysis for SII and SIRI in determining metabolic health status

	AUC	S.E.	Cut-off	Sensitivity	95% CI	Specificity
*SII*	0,854	0,04	146.4	82	0,770-0,962	72
*SIRI*	0,675	0,06	1,25	72	0,727-0,893	64

ROC, receiver operating characteristic; SII, systemic immune-inflammation index; SIRI, systemic immune-inflammation response index; AUC, area under curve; S.E., standard error; CI, confidence interval

After adjusting for age, sex, pubertal stage, and BMI SDS, the associations of both SII and SIRI with metabolic health status were attenuated; however, the overall trend persisted. For SII, the difference between metabolically healthy and unhealthy obese children showed reduced yet borderline significance (*p* = 0.056), indicating that developmental factors partially contribute to inflammatory variation while not fully eliminating the relationship. Similarly, SIRI demonstrated a weakened but still directional association after adjustment (*p* = 0.091), consistent with the pattern observed in the unadjusted analyses. Pubertal stage and sex did not independently influence either SII or SIRI (all *p* > 0.40), and no significant interaction was observed between metabolic health status and pubertal stage. These adjusted findings suggest that although developmental factors dilute the magnitude of group differences, systemic inflammation continues to show a tendency toward higher levels in metabolically unhealthy obese children.

Multivariable regression analysis demonstrated a significant positive association between the SII and key cardiometabolic risk markers. Higher SII values were associated with increased HOMA-IR (r² = 0.375, 95% CI: 0.225–0.525, *p* = 0.006), indicating a link between systemic inflammation and insulin resistance. Similarly, SII showed a strong relationship with the TG/HDL ratio (r² = 0.436, 95% CI: 0.284–0.595, *p* = 0.004), a known marker of atherogenic dyslipidemia. A significant association was also observed between SII and non-HDL cholesterol (r² = 0.239, 95% CI: 0.086–0.385, *p* = 0.01). These results suggest that elevated SII values reflect a greater burden of cardiometabolic risk in obese children (Table 4).

**Table 4:** Association between systemic inflammation index (SII) and cardiovascular risk parameters

SII	*r* ^2^	95%CI	*p*-value
*HOMA-IR*	0,375	0,225-0.525	***0***,***006***
*TG/HDL*	0,436	0,284-0,595	***0***,***004***
*Non-HDL*	0,239	0,086 − 0,385	***0***,***01***

SII, systemic immune-inflammation index; CI, confidence interval; HOMA-IR, homeostasis model assessment of ınsulin resistance; TG: HDL-C, triglyceride-to-high-density lipoprotein cholesterol; non-HDL-C, non-high-density lipoprotein cholesterol

When inflammatory indices were evaluated across pubertal stages within each metabolic phenotype (MUO/MHO), no significant differences were observed among prepubertal, pubertal, and postpubertal children in the MUO group (all *p* > 0.36). Although inflammatory markers also showed no significant variation across pubertal stages in the MHO group, PLR demonstrated a modest but statistically significant increase with advancing pubertal stage (*p* = 0.032). Notably, SII and SIRI tended to be higher in postpubertal MUO participants, although these differences did not reach statistical significance.

## Discussion

In this study, we comprehensively examined the relationship between metabolic health phenotypes and inflammatory profiles in childhood obesity. Our findings suggest that children with MUO may tend to higher inflammatory activity compared with their metabolically healthy counterparts. These observations indicate that metabolic impairment in obese children may not be fully accounted for by adiposity or conventional anthropometric parameters alone, and that systemic inflammation could represent a contributing biological component in distinguishing metabolic phenotypes. Overall, the pattern observed in our study indicates a possible increase in inflammatory activity among MUO children, although the cross-sectional design limits firm inferences regarding causality.

Low-grade inflammation originating from adipose tissue is recognized as one of the central biological mechanisms linking obesity to metabolic deterioration [3, 13, 18–27]. Chronic energy excess promotes adipocyte hypertrophy and induces homeostatic stress, which in turn triggers an adaptive inflammatory response through chemokine release. Once the expansion capacity of adipose tissue is exceeded, this response evolves into a chronic, self-sustaining inflammatory cycle [3, 13, 18, 19, 23]. Numerous studies have shown that increasing adipose tissue dysfunction is accompanied by higher circulating levels of pro-inflammatory mediators, which are closely related to various components of the metabolic syndrome [11, 16, 19, 24–25]. In our study, the relatively higher inflammatory indices observed in the MUO group align with these proposed mechanisms and suggest that low-grade inflammation may contribute to metabolic health differences in obese children.

Our study also found that WBC, neutrophil, and monocyte levels were significantly higher in the MUO group, aligning with previous pediatric research describing low-grade inflammatory activity in obesity. Anık et al. reported progressively elevated WBC, neutrophil, lymphocyte, and monocyte counts across morbidly obese, obese, overweight, and healthy-weight children [23], while different studies similarly linked increased neutrophil and monocyte counts to insulin resistance and dyslipidemia in obese adolescents [9, 11, 16, 18, 19, 23].

Integrated with this body of evidence, our findings suggest that the MUO phenotype may be accompanied by a relatively higher inflammatory tendency. Nonetheless, because these associations are derived from observational data, both in our study and in prior work, conclusions regarding causality should be drawn with appropriate caution.

Recent studies have shown that hematological inflammatory markers are gaining increasing clinical importance in the diagnosis and prognosis of chronic diseases [13, 15, 27–29]. The strong relationship between inflammation and alterations in peripheral blood cell counts has drawn attention not only to classical inflammatory markers (CRP, leukocyte count, neutrophil level), but also to composite indices derived from complete blood counts—such as NLR, PLR, SII, and SIRI—which have emerged as more sensitive and specific indicators [15, 23, 24, 31–36].

In our study, the high AUC value (0.854) observed for SII in distinguishing metabolic phenotypes may be attributed to its ability to reflect both thrombocyte and neutrophil activation simultaneously. In adult cohorts, SII has been repeatedly shown to outperform NLR and PLR in determining cardiovascular mortality and atherosclerotic burden [30, 31]. In the National Health and Nutrition Examination Survey (NHANES) with 42,785 participants, elevated SII and SIRI levels were associated with both cardiovascular and all-cause mortality [32]. In addition, a study including nearly 14,000 participants reported that SII and SIRI were significantly elevated in obese individuals, supporting the notion that inflammatory load may represent an important component in obesity management [24].

In the pediatric population, the available data are more limited. Huang et al. reported that SII was associated with hepatic steatosis and insulin resistance in obese children [17]. Luo and colleagues showed that SII and SIRI were positively correlated with BMI in children and adolescents, suggesting that these indices may serve as potential markers for assessing systemic inflammation in pediatric obesity [36]. In another study, Öztürk et al. found higher NLR, PLR, and SII levels in children with metabolic syndrome [37]. Similarly, Nicoara et al. highlighted that SII presented a higher AUC value compared with other CBC-derived indices for distinguishing metabolic syndrome in obese children [11].

In our study, the strong associations of SII with HOMA-IR, TG/HDL, and non-HDL suggest that inflammation may represent both a potential contributor to and a reflection of metabolic impairment. Consistent with the literature, TNF-α and IL-6 are known to impair hepatic insulin signaling; monocyte-derived cytokines contribute to dyslipidemia by increasing lipolysis; and platelet activation has been associated with early endothelial dysfunction (15,16). The ability of SII to reflect these mechanisms supports the biological plausibility of our findings. Unlike studies focused on metabolic syndrome, research evaluating the association between metabolic health status (MHO–MUO) and inflammatory markers is limited. Our study addresses this gap and demonstrates that SII and SIRI have meaningful diagnostic value for distinguishing metabolically unhealthy obese children. According to the results of this study, which is the first of its kind in the literature, SII and SIRI have diagnostic value in distinguishing metabolically unhealthy individuals among obese children. Additionally, both parameters were positively correlated with cardiovascular metabolic biomarkers.

In addition to group differences, the regression analyses provided further insight into the metabolic correlates of systemic inflammation. Higher SII values were modestly but consistently associated with key cardiometabolic markers, including insulin resistance (HOMA-IR), the TG/HDL ratio, and non-HDL cholesterol. Although the effect sizes were moderate (r² = 0.24–0.44), these relationships suggest that inflammatory activation may track with early perturbations in glucose–lipid homeostasis. Similar associations between neutrophil- and platelet-driven inflammatory indices and insulin resistance have been reported in both adult and pediatric cohorts, supporting the concept that metabolic inflammation and cardiometabolic dysregulation evolve in parallel rather than in isolation. Luo et al. examined the relationship between SII and glucose–insulin dynamics and reported positive correlations between SII and fasting glucose, fasting insulin, and HOMA-IR in regression analyses. In the same study, individuals with higher SII values demonstrated a modestly increased likelihood of prediabetes and insulin resistance (approximately 1.1- to 1.3-fold) compared with those with lower SII values [38]. In a large cohort of 1,201 obese children and adolescents, Genovesi et al. reported a high prevalence of metabolically unhealthy obesity (61%). MUO individuals were more often male, older, and pubertal, and exhibited significantly higher levels of uric acid, HOMA-IR, and waist-to-height ratio compared with their MHO peers. Notably, all three parameters were identified as independent predictors of MUO, with odds ratios ranging from 1.03 to 1.41. The authors also observed that approximately 15% of children classified as MHO nevertheless demonstrated upper-quartile values for uric acid, HOMA-IR, and waist-to-height ratio—highlighting that metabolic risk can be present even in seemingly “healthy” obese phenotypes [39]. Given the cross-sectional nature of our data, these correlations cannot establish directionality; however, they lend biologic plausibility to the proposition that SII reflects a composite burden of inflammatory and metabolic stress in obese children.

Although different studies have applied various criteria to define MUO, the reported prevalence of the MHO phenotype ranges widely (20–68%). The literature generally indicates that the MHO group tends to be younger, more common in girls, and characterized by lower BMI SDS values [5, 7, 8, 40, 41]. However, it has also been emphasized that these characteristics are not consistent across all studies. In our study, 54.4% of the participants were classified as MHO, yet no significant differences were observed between the groups with respect to age or sex. Conversely, the higher frequency of morbid obesity and elevated BMI SDS values in the MUO group supports the notion that metabolic impairment is closely related to the severity of adiposity. There is growing evidence suggesting that the MHO phenotype may not represent a stable condition. Longitudinal studies have shown that a substantial proportion of MHO children transition to the MUO phenotype over time. For example, some cohorts have reported that 76% of individuals classified as MHO later converted to MUO, whereas another study demonstrated that 70% lost their metabolically healthy status after 7.7 years. In the longest follow-up study, with an average duration of 24 years, only 13% of individuals who were MHO in childhood remained metabolically healthy in adulthood [35]. These findings underscore the importance of early identification of shifts in metabolic health to appropriately target at-risk children and to develop preventive strategies. The potential ability of inflammatory markers to predict this transition may provide an important contribution to early risk stratification [36]. This study analyzed complementary laboratory data related to the metabolic health status of obese children. To the best of our knowledge, this is the first study to examine the relationship between SII and SIRI with cardiometabolic risk biomarkers in metabolically unhealthy obese children. This provides confirmatory evidence that inflammatory processes begin early in metabolically unhealthy obese children and emphasises the role of these indices as additional measures of cardiometabolic imbalance.

These findings are clinically significant, particularly in pre-hospital settings, as they could improve the routine care and differentiation of metabolically unhealthy obese children.

In our study, the attenuation—but not complete disappearance—of the association between inflammation and metabolic health after adjustment for age, sex, and pubertal stage suggests that, despite the strong biological influence of puberty on inflammatory markers, MUO children may still exhibit an ongoing tendency toward higher inflammatory activity. Puberty is recognized as a physiological period marked by increased inflammation due to GH/IGF-1 axis activation, rising sex steroid concentrations, and immune system remodeling [41]. For this reason, puberty constitutes an important biological variable that contributes to variability in inflammatory indices.

Supporting evidence from the literature further reinforces this concept. In a large adolescent cohort, Zhang et al. demonstrated that individuals in the highest SII quartile exhibited more than a three-fold greater risk of central obesity compared with those in the lowest quartile (OR = 3.07; 95% CI: 2.45–3.87). Longitudinally, adolescents with the highest SII levels also showed a significantly increased risk of developing central obesity over time (RR = 1.83; 95% CI: 1.18–2.83). Additionally, pubertal onset has been reported to approximately double the risk of transitioning from MHO to MUO, whereas progression to mid-late puberty nearly tripled the likelihood of transitioning from MUO to MHO [42].

Within this context, the persistence of borderline significance for SII in our study (*p* = 0.056) indicates that, although developmental factors diminish the strength of the association, MUO children may still display a tendency toward elevated inflammatory activity. This observation suggests that the inflammatory load accompanying the metabolically unhealthy phenotype in childhood cannot be fully explained by pubertal or developmental changes alone, and may represent an additional component of metabolic dysregulation.

A related investigation examining the interplay between puberty, MetS, obesity, and inflammation further demonstrated that obese children with higher prepubertal tPAI concentrations had a 19% greater likelihood of developing MetS during puberty [43]. Although assessment of cardiometabolic and inflammatory status in prepubertal obese children is emphasized as critical for preventing later comorbidities, our study was not able to establish pubertal transition thresholds for systemic inflammatory markers.

Despite repeated reports in the literature highlighting a strong link between central obesity and MUO [39, 44], we did not observe significant differences in waist circumference or waist-to-height ratio in our cohort. This finding raises the possibility that metabolic deterioration in children may precede overt changes in fat distribution, that simple anthropometric measures may insufficiently reflect visceral adiposity, and that pubertal biological variability might mask differences in central fat accumulation.

Taken together, these observations suggest that the dynamic interplay among adipose tissue expandability, low-grade inflammation, and pubertal biology may play a pivotal role in shaping MHO–MUO transitions during childhood and adolescence.

A major strength of this study is the relatively large sample size from a single tertiary center, which ensured standardized assessments and consistent laboratory methodology. The use of pre-treatment data also strengthens the validity of the inflammatory and metabolic measurements. Assessing multiple hematological indices together with cardiometabolic markers provides a broad, practical perspective on the potential clinical utility of these low-cost biomarkers.

This study also has limitations. Its cross-sectional design precludes causal inference, restricting conclusions to associations only. Although a single-center setting reduces measurement variability, it may limit generalizability to other populations. In addition, we were unable to compare SII and SIRI with more specific inflammatory biomarkers (e.g., cytokines or adipokines), which limits insight into their relationship with established markers of metabolic inflammation. Finally, despite adjustment for pubertal stage, residual confounding related to the dynamic nature of puberty cannot be fully excluded.

## Conclusion

This study demonstrates that hematological inflammation indices—particularly SII and, to a lesser degree, SIRI—may serve as simple, low-cost, and accessible markers for identifying metabolically unhealthy obesity in children. By jointly evaluating metabolic phenotypes and cardiometabolic risk markers, our work offers novel pediatric evidence on the clinical relevance of these indices.

Although the cross-sectional design limits causal interpretation, the consistent associations observed with key metabolic risk indicators suggest that systemic inflammation may accompany early metabolic deterioration. Importantly, these patterns persisted after adjustment for age, sex, and pubertal stage, indicating that developmental factors alone do not fully explain the inflammatory differences between MHO and MUO.

Given their wide availability and ease of use, SII and SIRI may provide practical complementary tools in primary care for early risk stratification. Future multi-center longitudinal studies incorporating detailed body-composition data and validated inflammatory markers are needed to clarify temporal relationships and determine whether SII and SIRI can predict transitions between MHO and MUO or long-term cardiometabolic outcomes.

## Data Availability

All materials related to this article, including data sets and additional information, are available upon request.
